# Effect of Antifreeze Glycopeptides on the Quality and Microstructure of Frozen Lamb Meatballs

**DOI:** 10.3390/metabo15030202

**Published:** 2025-03-13

**Authors:** Rong Dong, Shengkun Yan, Guoqiang Wang, Pei Wang

**Affiliations:** 1 Agricultural Mechanization Institute, Xinjiang Academy of Agricultural Sciences, Urumqi 830091, China; 15699172240@163.com (R.D.); 1811111230@mail.sit.edu.cn (S.Y.); 2 College of Food Science and Technology, Nanjing Agricultural University, Nanjing 210095, China; ddd123450206@163.com

**Keywords:** antifreeze glycopeptides, lamb meatballs, water distribution, microstructure, untargeted metabolomics

## Abstract

This study explored the protective effects of antifreeze glycopeptide and alginate on the quality of −18 °C frozen lamb meatballs across various storage periods. Methods: Measurements of volatile salt nitrogen (TVB-N), thiobarbituric acid (TBARS), water retention, water distribution, microstructure, and metabolite changes were taken in the lamb meatballs. Results: The results showed that the addition of antifreeze glycopeptides (AFGs) significantly preserved the quality characteristics of lamb meatballs. In particular, the 0.30% antifreeze glycopeptide demonstrated the strongest protective effect on water retention and metabolites during freezing. The ice crystal area within the microstructure of lamb meatballs with added antifreeze glycopeptides was markedly reduced compared to the others after 14 days of freezing (*p* < 0.05). Additionally, AFGs lessened the lipid oxidation reaction and prolonged the oxidation time of lamb after 28 days of freezing. Conclusion: In summary, AFGs beneficially affected the quality of frozen lamb meatballs and are a potential, safe, and efficient cryoprotectant.

## 1. Introduction

Mutton, a staple in the human diet, is celebrated globally for its distinctive flavor and nutritional benefits [1]. A diverse array of mutton products, such as deli meats, canned mutton, and meatballs, has not only expanded dietary options but also catered to various consumption scenarios and needs [2]. In particular, pre-prepared mutton meatballs, which are convenient and quick, have been extensively researched and utilized in the food industry. However, maintaining the quality of pre-made meatballs and other mutton products during freezing and storage remains a challenge. Issues include quality degradation due to repeated freezing and thawing, characterized by reductions in pH and water retention, an increase in TBARS and TVB-N values, a rise in the total colony count, and a general decline in tenderness [3,4,5]. Moreover, the method of freezing markedly influences the quality, water distribution, microstructure, and flavor profile of long-term frozen mutton [6,7,8]. To mitigate these issues, cryoprotectants are often employed. These are substances that help preserve the quality of frozen products by reducing ice crystal formation and delaying oxidative processes. Traditional synthetic cryoprotectants, such as glycerol and dimethyl sulfoxide (DMSO), have been widely used; however, there is increasing interest in more effective natural alternatives, such as antifreeze glycopeptides, antifreeze proteins, and polysaccharides.

Antifreeze glycopeptides (AFGs), naturally occurring biomolecules, play a crucial role in protecting cells and tissues at low temperatures by managing the size and shape of ice crystals to prevent ice growth. The natural sources of AFGs include certain cold-adapted organisms such as fish, plants, and insects that thrive in freezing environments. The formation mechanism of AFGs involves the biosynthesis of specific glycoproteins that interact with ice crystals, inhibiting their growth and recrystallization through adsorption inhibition processes [9,10]. AFGs are extensively utilized in various domains, such as biomedicine and food technology, primarily due to their ability to induce significant thermal hysteresis and inhibit recrystallization [11,12]. Antifreeze glycopeptides significantly reduce ice crystal formation and the associated tissue damage in frozen meat products by lowering the freezing point of solutions without affecting the melting point, thereby preserving the quality and integrity of the meat [13,14].

The objective of this study was to examine the impact of antifreeze glycopeptides on lamb meatballs. The experimental treatments included a saline solution (CK), an antifreeze glycopeptide (AFG) solution, and an alginate (ALG) solution, frozen for 0, 7, 14 and 28 days, respectively. Utilizing advanced techniques such as thiobarbituric acid reactive substances (TBARSs), total volatile base nitrogen (TVB-N), low-field nuclear magnetic resonance (LF-NMR), scanning electron microscopy (SEM), and non-targeted metabolomics (LC/MS), this study assessed the moisture distribution, microstructure, and metabolite alterations in lamb meatballs. The primary purpose of this study is to provide a scientific basis and a novel strategy for quality control in the freezing and preservation of lamb meatballs. By investigating the cryoprotective mechanisms of AFGs, this work aims to deepen our understanding of how AFGs mitigate quality deterioration in frozen lamb products, particularly through their ability to regulate ice crystal formation, stabilize moisture distribution, and reduce oxidative damage. The findings of this study are expected to contribute to the development of effective cryoprotectants for the meat industry, offering practical solutions that improve the texture, nutritional value, and shelf life of frozen lamb products.

## 2. Materials and Methods

### 2.1. Test Materials and Reagents

Fresh lamb meat was sourced from a local meat market in Urumqi, Xinjiang, China, within 24 h of slaughter. Alginose (food grade) was purchased from Hayashibara Co. Ltd., Okayama, Japan, and antifreeze glycopeptides were produced in-house by the laboratory (the specific methods are as follows). Reagents such as sodium hydroxide (NaOH), 4,6-dihydroxy-2-mercaptopyrimidine (TBA), trichloroacetic acid-HCl (TCA-HCl), and butylated hydroxytoluene (BHT) were acquired from Aladdin (Shanghai, China).

Preparation of walnut meal peptide: Defatted walnut meal powder was dispersed in deionized water at a ratio of 1:10 (*w*/*v*) and its pH was adjusted to 8.0 with 1 M NaOH; it was then stirred at room temperature for 2 h and centrifuged at 10,000× *g* for 30 min at 4 °C. The supernatant was taken and the pH was adjusted again to 4.5 with 1 M HCl, and the supernatant was then allowed to stand for 2 h at room temperature (approximately 25 °C) before centrifugation for 30 min. After the precipitate was washed with water, the pH was adjusted to pH 7 with 1 M NaOH and freeze-dried to obtain walnut meal protein. Walnut meal proteins were dispersed in deionized water, configured to a substrate concentration of 5%, and incubated at the optimal temperature of 65 °C for the alkaline protease (Beijing Solarbio Science & Technology Co., Ltd., Daxing District, Beijing, China), with an enzyme addition of 2% (E/S, *w*/*w*) (in terms of walnut protein content). All reactions were carried out at an optimum pH of 8.5 for the alkaline protease; the pH was then adjusted to neutral after the enzymatic digestion, and the alkaline protease was inactivated by boiling water bath, cooled by ice water, centrifuged to obtain the supernatant, and freeze-dried to produce walnut meal peptide.

The preparation of polysaccharides: Wheat bran was inactivated in an oven at 130 °C for 150 min. After inactivation, it was ground into powder and passed through a 30-mesh sieve. Then, 100 g of wheat bran was weighed and dispersed in 750 mL of deionized water at pH 6.6. After heating to 90 °C, high-temperature-resistant α-amylase (0.6 g, 120 KNU-T/g, Beijing Solarbio Science & Technology Co., Ltd., Daxing District, Beijing, China) was added, and the reaction was stirred for 2 h. At the end of the stirring process, the pH of the solution was adjusted to 7.5, and Protex 14 L (5 mL/mg, 160 U/g) was added, after which the reaction was carried out for 5 h at 65 °C. After the hydrolysis of starch and protein, the wheat bran suspension was placed in an oven at 100 °C for 150 min to inactivate the enzyme, and then it was ground and sieved through a 30-mesh sieve. After hydrolyzing the starch and protein, the bran suspension was heated at 100 °C for 15 min to inactivate the enzyme. The digested bran was filtered, washed three times with water and dried. A solution containing 7% (*w*/*v*) sodium hydroxide and 12% (*w*/*v*) urea was cooled down to −12.6 °C in a −20 °C refrigerator until ice crystals began to form. Bran stripped of protein and starch was added to the above solution to form a 0.5% (*w*/*w*) suspension. The mixture was stirred magnetically in a water bath at a temperature setting of 25 °C for 12 h. The mixture was then cooled to −12.6 °C in a refrigerator at −20 °C, after which it was stirred at 25 °C for another 12 h. The mixture was then centrifuged for 10 min at a temperature of 4 °C at 12,100× *g*. An ethanol solution was added gradually to the centrifuged supernatant, after which it was subjected to sequential gradient ethanol (60%) precipitation. The resulting precipitates were redissolved in water, dialyzed (dialysis bags) and lyophilized after the addition of the dissolved water. The ratio of the amount of sugar obtained at each alcohol precipitation concentration to the de-proteinized and de-amylated bran was the yield at each concentration. The content of polysaccharides extracted with urea and alkali ranged from 52.33 to 0.13%.

The formulation involved a blend of 1% walnut meal peptide and 1% bran polysaccharide by weight per volume. The pH was adjusted to 8.0 using a 0.1 M sodium hydroxide solution. This mixture was subjected to standard wet heating at 60 °C for 24 h in a water bath, followed by cooling to halt the reaction, and subsequently dialyzed for 24 h. The samples were freeze-dried to obtain antifreeze glycopeptides.

### 2.2. Sample Preparation

Fresh lamb meat was transported to the laboratory and ground into meatballs. The samples were divided into nine groups and immersed in 0.9% saline (CK), 0.3% (*w*/*v*) antifreeze glycopeptide (AFG) solution, and 4% (*w*/*v*) alginate (ALG) solution for 2 h. Surface water was removed from the lamb meatballs using filter paper, after which they were placed in sterile bags, sealed, and frozen at −18 °C for 0, 7, 14, and 28 days. FCK, as indicated by metabolomics analysis, represents the frozen 0 day samples. Pre-treatment was conducted according to the method designated for each group, with three sets of replicates performed for each treatment.

### 2.3. TBARS Measurement

A 2 g sample of defrosted lamb meatballs was ground and combined with 1% TBA (3 mL) and 2.5% TCA-HCl (17 mL). In a boiling water bath (made by Kewei Yongxing, Chaoyang District, Beijing, China) set at 100 °C for 30 min, the mixture was subjected to butylated hydroxytoluene (BHT, 0.19 M, 0.5 mL) before being cooled to 25 °C. The mixture was then vortexed for 1 min after adding 4 mL of chloroform to the cooled suspension. After 20 min of centrifugation at 3000 rpm, the absorbance at 532 nm was measured using a device manufactured by Youke Instrument Co., China [15]. Here is how TBARS values are presented:

$$\mathrm{TBARS}\;(\mathrm{mg}/\mathrm{kg})=\frac{A_{532}}{Ws}\times 9.48$$

Here, A_532_ is the absorbance of the measured solution at 532 nm; Ws is the weight of the sample (g); and 9.48 is a constant derived from the dilution factor and molar extinction coefficient of the TBA reaction product (152,000 M^−1^·cm^−1^).

### 2.4. TVB-N Determination

Initially, the thawed specimens were finely chopped and amalgamated with 100 mL of distilled water. After a 30 min immersion, the samples underwent centrifugation at 3000 rpm for 10 min. Subsequently, 1 mL of the resulting supernatant and 1 mL of a saturated solution of potassium carbonate were introduced into Conway’s external mixture. Following this, 1 mL of a 10 mM boric acid solution and 1 mL of a mixed indicator—comprising 1 g/L of methyl red and 5 mL of bromocresol green in ethanol—were added to the inner chamber of the Conway dish. After a 120 min incubation at 37 °C, the solution was titrated with 10 mM of hydrochloric acid until a light pink hue was achieved. The equation for TVB-N values (mgN/100 g) was applied at this stage:

$$\mathrm{TVB}-N=\frac{{[(V}_{1}-V_{2})\times C\times 0.14\times d\times 100]}{m}$$

Here, V_1_ is the titration volume of the test sample; V_2_ is the titration volume of the blank; m is the weight of the sample; C is the hydrochloric acid concentration; and d is the dilution factor.

### 2.5. Transverse Relaxation Time and Nuclear Magnetic Resonance Imaging Measurements

To prevent dehydration, approximately 10 g of sample was encased in plastic wrap and placed within a nuclear magnetic resonance (NMR) tube, which featured a 70 mm coil diameter. Measurements were conducted at a proton resonance frequency of 21 MHz, within a magnetic field of 0.55 T, and at a controlled temperature of 32 °C. The transverse relaxation time, T_2_, of the lamb samples was assessed using a Carr–Purcell–Meiboom–Gill (CPMG) sequence, with the primary settings being as follows: an RF signal frequency offset (O1) of 171,005.93 Hz, a total of 400,004 sampling points (TD), a 90° pulse width (P) of 17.00 µs, a 180° pulse width (P) of 32.00 µs, a sampling frequency (SW) of 100 kHz, an echo time (TE) of 0.5 ms, a sampling wait time (Tw) of 4000 ms, an RF delay (RFD) of 0.08 ms, an analog gain (RG1) of 20.0 dB, a digital gain (DRG1) of 6 dB, two accumulations (NS), and 8000 echoes (NECH). Signals were acquired using NMRAS analysis software (NMRPipe 9.0), and the data were normalized after inversion. Magnetic resonance imaging (MRI) was utilized to determine the proton density profile of lamb, and the resulting proton density map was analyzed to determine the image’s gray level by Image Evaluation software (R2023a).

### 2.6. Cryo-SEM

Lamb meatball samples were added dropwise onto a special sample tray for cryo-scanning. The sample tray was then rapidly immersed in liquid nitrogen slush for 30 s before being relocated to a sample preparation chamber where it underwent sublimation gold plating in a vacuum environment using a cryopreparation transfer system. This process was aimed at preserving the sample structure, ensuring that the water became glassy rather than crystallizing during the curing process, thus preventing volume expansion and damage to the sample’s original structure. High-pressure freezing methods and liquid nitrogen slurry rapid freezing methods were typically employed for freezing and fixing the sample. Afterward, the sample was fractured by a special device in a vacuum and at low temperatures (−100 °C) to expose a fresh cross-section. Sublimation of the samples was carried out at −90 °C for 10 min, followed by sputter coating with gold at a 10 mA current for 30 to 60 s. Subsequently, the samples were transferred to the SEM sample chamber, where a cold stage temperature of −140 °C was maintained and an accelerating voltage of 5 kV was employed. The samples, maintained in a cryogenic state, were transferred to the cold stage inside the SEM sample chamber using a cryo-transfer system, with temperatures maintained as low as −140 °C or lower. Observation and imaging were conducted in such a cryogenic environment. The surface structure and internal structural information of the lamb meatballs were observed under the cryo-SEM, and high-resolution images were acquired.

### 2.7. Non-Targeted Metabolomics Assays

The necessary quantity of the sample was accurately dispensed into a 2 mL centrifuge tube, and 1000 µL of tissue extract consisting of 75% methanol and 25% water (with a 9:1 methanol: chloroform ratio) was added, along with steel beads. The sample was placed in a tissue grinder and homogenized at 50 Hz for 60 s, with the homogenization process being repeated once more. The sample was then exposed to sonication for 30 min at ambient temperature, followed by chilling on ice for an additional 30 min. Subsequently, the sample was centrifuged at 12,000 rpm at 4 °C for 10 min. The resulting supernatant was transferred into a new centrifuge tube for concentration and drying. The dried sample was reconstituted with 200 µL of a 50% acetonitrile solution and loaded into a vial for LC-MS analysis.

Chromatographic separation was performed on an ACQUITY UPLC^®^ HSS T3 (Waters Corporation, Milford, United States)column (2.1 × 100 mm, 1.8 µm) sourced from Waters, Milford, MA, USA. The column was maintained at a temperature of 40 °C, with a flow rate of 0.3 mL/min, and 2 µL of the sample was injected. In positive ionization mode, the mobile phases consisted of 0.1% formic acid in both acetonitrile (B2) and water (A2). The gradient elution profile started with 10% B2 for the first min, increased to 98% B2 over the next 4 min, was sustained at 98% for 1.5 min, then quickly reverted to 10% B2 within 0.1 min, and was maintained at 10% for the final 1.4 min. In negative ionization mode, the mobile phases of acetonitrile (B3) and 5 mM of ammonium formate in water (A3) followed a similar gradient: starting at 10% B3, increasing to 98% over 4 min, holding at 98% for 1.5 min, decreasing to 10% B3 in 0.1 min, and finally stabilizing at 10% B3 for the last 1.4 min.

The Thermo Orbitrap Exploris 120 mass detector (Thermo Fisher Scientific, Waltham, MA, USA) equipped with an electrospray ionization source (ESI) was utilized for data collection. Data were acquired separately in positive and negative ion modes. The positive ion spray voltage was set to 3.50 kV, while the negative ion spray voltage was set to −2.50 kV. The sheath gas was set to 40 arb, and the auxiliary gas was set to 10 arb. The capillary temperature was maintained at 325 °C. A primary full scan was performed at a resolution of 60,000, and the primary ion scan was conducted within the *m/z* range of 100 to 1000. High-resolution secondary cleavage was achieved using HCD with a collision energy of 30% and a secondary resolution of 15,000. The first four ions of the acquired signal were selected for fragmentation. Dynamic exclusion was applied to remove unnecessary MS/MS information. Raw mass spectra data files were converted to the mzXML file format using the MSConvert tool within the Proteowizard package (version 3.0.8789). Peak detection, peak filtering, and peak alignment were performed using the R XCMS software package with parameters set to bw = 2, ppm = 15, peakwidth = c (5, 30), mzwid = 0.015, mzdiff = 0.01, and method = “centWave”. A list of substances for quantification was generated.

Database Search and Comparison: Metabolites were identified by searching and comparing against multiple authoritative databases, including HMDB, MassBank, LipidMaps, mzCloud, KEGG, and a self-built metabolite standard database [15]. Cross-validation using multiple databases was performed to enhance the confidence of annotation. MS/MS Spectral Matching: The experimentally detected MS/MS spectra were compared with reference spectra from the databases. Matching was based on the mass-to-charge ratio (*m/z*) and relative intensity of the fragment ions. A match score threshold of >80% was applied, and the consistency of the isotopic distribution and ion mode (positive/negative) was also considered [16,17]. Confidence Level of Annotation: According to the Metabolomics Standards Initiative [16], the metabolite annotations in this study belong to Level 2 (identification based on database matching). Screening of Differential Metabolites: Based on the results of the primary differential analysis (e.g., *p* value and VIP threshold), secondary differential metabolites were screened to focus on statistically significant metabolites.

### 2.8. Data Processing

Trials were replicated three times for all experiments, with the results presented as mean ±standard deviation (SD). Statistical significance was assessed using one-way ANOVA with SPSS 23.0, considering *p* < 0.05 as indicative of significant differences among treatment means. Graphical representations were produced using Origin 2021.

## 3. Results and Discussion

### 3.1. TBARS and TVB-N Analysis of Lamb Meatballs During Storage

**Figure 1:**
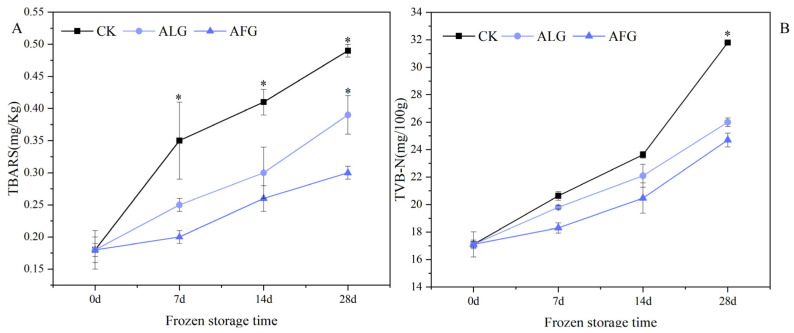
The variations in TBARS and TVB-N levels throughout the storage of black (CK), the antifreeze glycopeptide (AFG) solution, and the alginate (ALG) solution. (**A**): TBARS measurements in lamb meatballs following various treatments; (**B**): TVB-N measurements in lamb meatballs following various treatments. * Represents significant differences between treatments at the same storage time.

The TBARS value reflects the mass fraction of malondialdehyde (MDA), the final oxidation product within the system. In lipid oxidation processes, the presence of MDA correlates directly with the extent of fat degradation [18,19]. Illustrated in Figure 1A, the TBARS values in lamb meatballs progressively increased over the freezing period. In comparison, the TBARS levels in the CK group were significantly elevated compared to those in the other two experimental groups (*p* < 0.05), with the AFG group exhibiting markedly lower levels (*p* < 0.05). In particular, the TBARS measurements in the CK lamb meatballs surged from an initial 0.18 mg/kg to 0.49 mg/kg, while those in the AFG samples rose from 0.18 mg/kg to 0.30 mg/kg. The rate of TBARS value increase in the ALG group was intermediate, with a 5.26% higher increment compared to the AFG group and a 22.3% lower increment relative to the CK group at 28 days. This illustrates the effectiveness of AFG samples in curtailing the oxidation of polyunsaturated fatty acids and thus moderating the rise in TBARS values [20,21,22]. Alginose possesses a higher glass transition temperature, which increases the likelihood of a glassy state being formed. In this state, the rate of chemical reactions within the glass matrix is reduced, thereby effectively inhibiting fat auto-oxidation.

TVB-N is a metric used to assess meat freshness, where elevated levels typically indicate a reduced nutritional quality and potential spoilage [23,24]. As depicted in Figure 1B, the TVB-N levels in lamb meatballs incrementally increased throughout the storage period, with a modest rise from day 0 to day 14, followed by a marked escalation from day 14 to day 28. The TVB-N values of the CK group were significantly higher than those of the other treatments (*p* < 0.05), indicating a greater extent of deterioration. In contrast, the application of antifreeze peptides and alginate significantly slowed the progression of deterioration in the lamb meatballs, with antifreeze peptides exhibiting a more pronounced effect.

### 3.2. Moisture Migration and Visualization During Storage of Lamb Meatballs

**Figure 2:**
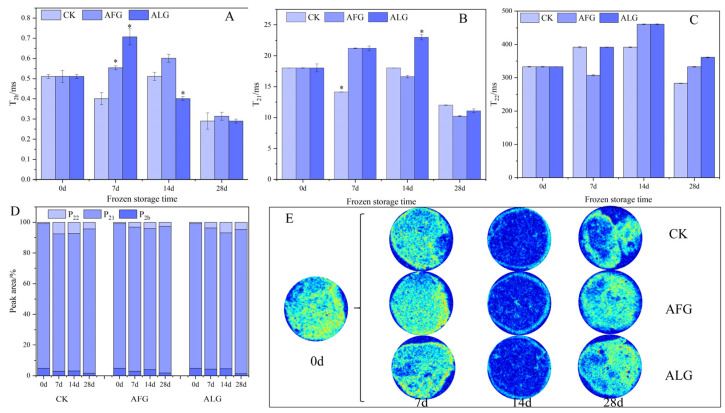
The moisture migration and phase changes during the storage of lamb meatballs. (**A**): T_2b_ values in lamb meatballs over different storage durations; (**B**): T_21_ values in lamb meatballs over different storage durations; (**C**): T_22_ values in lamb meatballs over different storage durations; (**D**): moisture peak area ratio in lamb meatballs over different storage durations; (**E**): moisture phase changes in lamb meatballs during storage.

The LF-NMR is utilized as a non-destructive analytical method to evaluate the state of moisture in meat [25]. In this methodology, the T_2_ relaxation time is utilized as the principal metric for discerning between various states of moisture [26]. T_2b_ (0–10 ms) designates bound water, which is firmly attached to macromolecular substances [27]. The quantity of bound water remains consistent and is not influenced by alterations in the protein structure or net charge [28]. Notably, the T_2b_ values were significantly elevated in the treatment group compared to the control group after 7 days of storage, with the ALG lamb displaying the most pronounced increase in T_2b_ (Figure 2A). At 14 and 28 days of storage, the AFG lamb showed the highest T_2b_, suggesting an increase in the proportion of bound water in mutton. Bound water forms a layer tightly bound to muscle protein molecules, possesses a low freezing point of around −40 °C, is difficult to dissociate and evaporate, and is resistant to structural and charge changes in muscle proteins [29,30]. Bound water does not directly influence muscle water retention as it is very tightly bound to proteins and cannot alter its binding state, even under severe external force [31]. A notable decrease in T_2b_ at 28 days of storage in lamb meatballs could be attributed to protein interactions that increase the hydrophobicity of protein surfaces, making it more difficult for proteins to bind water.

T_21_ generally denotes the less readily flowable water found within highly organized protein structures, including between actin/protomyosin thin filaments and myosin thick filaments. An increase in T_21_ indicates disrupted myogenin and the deterioration of water retention in lamb [32]. At 7 days of frozen storage, the treatment group did not demonstrate the anticipated freezing resistance. After 14 and 28 days of storage, the T_21_ levels in AFG lamb meatballs were found to be the lowest (Figure 2B). This suggests that AFGs are capable of modifying the direction of ice crystal formation, thereby mitigating protein oxidative degradation and curtailing water mobility. T_22_ is indicative of free water on the meat’s surface, accounting for a minor proportion [33]. T_22_ increased with prolonged storage and was equal for the AFG and ALG lamb at 14 days of storage. At 28 days, the T_22_ in the lamb decreased (Figure 2C), suggesting that freezing expands the internal spacing of muscle fibers, allowing water to migrate from the cell to the external environment more freely [34].

The figure shows that the ratio of the integral area to the total area, denoted as P_2b_, P_21_, and P_22_, mirrors the relative presence of various water components within the meatballs. The findings reveal that P_21_ correlates with the meat’s water-holding capacity. As the freezing duration increases, P_2b_ generally exhibits a decline. This trend may be attributed to the formation of irregular ice crystals, which intensify with the increasing severity of freezing in damaged muscle fibers. This process diminishes the water-holding capacity of myofibrillar proteins and leads to the outward migration of immobilized water, either transitioning into free water or associating with dissolved protein molecules to constitute bound water [35,36].

In summary, the observed increase in bound water (T_2b_) and reduction in less mobile water (T_21_) in the AFG-treated samples suggest that AFGs effectively mitigate ice crystal formation and protein degradation, thereby preserving the structural integrity of muscle fibers. This is directly linked to an improved texture quality, as reduced ice crystal damage minimizes muscle fiber disruption and maintains tenderness and juiciness in the meat. Furthermore, the stabilization of water mobility (T_22_) in AFG-treated samples indicates a slower migration of free water, which contributes to better water retention and reduced drip loss during thawing. These findings align with the hypothesis that AFGs enhance the quality of frozen meat by modulating ice crystal formation and water distribution, ultimately preserving the sensory and textural attributes of the final product.

### 3.3. Microstructure of Lamb Meatballs During Storage

**Figure 3:**
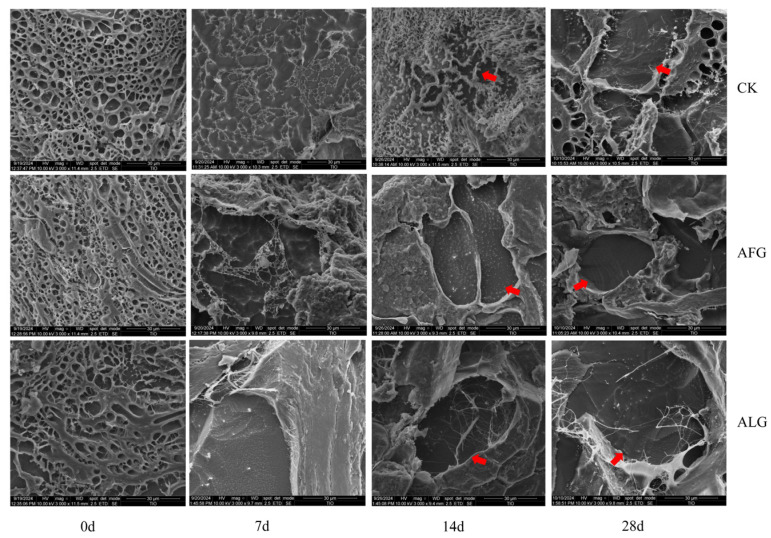
The microstructural changes in lamb meatballs over the storage period. Arrows indicate areas of ice crystal formation.

Alterations in microstructure demonstrate the condition of myofibrillar fibers within meat products, primarily driven by ice crystal accumulation and protein structure modifications throughout the frozen storage period [37]. Figure 3 illustrates that initially, at the onset of cryopreservation (0 day), all samples of lamb meat exhibited a uniformly aggregated structure characterized by densely packed myofibrillar fiber bundles and minimal voids within the intramuscular connective tissue. Subsequent to the initiation of cryopreservation, gaps emerged between the myofibrillar fibers, coinciding with the formation of ice crystals. By the seventh day of cryopreservation, pronounced ice crystal development was evident in the CK group, while the other groups displayed only an increase in the gaps within myofibrillar fibers. After 14 days of cryopreservation, significant ice crystal development was observed in the CK group. In contrast, both the AFG and ALG groups exhibited fine ice crystal development, with no significant difference noted between them. After 28 days of storage in a frozen state, myogenic fibers in the CK group exhibited severe deformation, and ice crystal formation was pronounced in the ALG group, with a tendency to form ice layers. Conversely, the AFG group maintained myofibrillar integrity more effectively than both the CK and ALG groups, despite a significant decrease in fiber integrity and an increase in ice crystal formation. These observations suggest that treatment with antifreeze glycopeptides was more effective. The freezing process disrupts the primus membrane, undermining the integrity of muscle tissue and disassembling its dense structure. Consequently, the myofibrillar fibers within these samples become disordered, fragmented, and break down into numerous smaller segments. This physical degradation primarily arises from the expansion and recrystallization of ice crystals within the muscle tissue over the course of storage. The cryopreserved lamb meat exhibited varying levels of tearing attributable to ice crystal formation, which ultimately led to the disintegration of muscle fibers [38].

The impact of variations in microstructure on the quality of the final product, particularly its textural quality, is profound and directly linked to the changes observed in myofibrillar fiber integrity and ice crystal formation. In the CK group, the pronounced development of large ice crystals and the severe deformation of myofibrillar fibers resulted in significant structural damage, leading to a loss of tenderness and increased toughness in the meat. This is consistent with the hypothesis that uncontrolled ice crystal growth disrupts muscle fiber networks, negatively affecting texture. In contrast, the AFG-treated samples exhibited finer ice crystals and the better preservation of myofibrillar integrity, which correlates with improved texture quality. The reduced ice crystal size and slower recrystallization in AFG-treated meat minimized muscle fiber disruption, maintaining tenderness and juiciness. This aligns with the objective of the work, which is to demonstrate that AFGs can enhance the quality of frozen meat by mitigating ice crystal-induced damage. Furthermore, the ALG group, while showing some improvement over the CK group, still exhibited ice layering and fiber degradation, indicating that AFGs are more effective in preserving microstructure and texture. The results of this study are consistent with the findings of [39,40], which findings underscore the critical role of microstructure in determining the sensory and textural attributes of frozen meat, supporting the hypothesis that AFG treatment can significantly improve the quality of frozen meat products by maintaining their structural integrity and minimizing texture degradation.

### 3.4. Metabolite Changes During Storage of Lamb Meatballs

**Figure 4:**
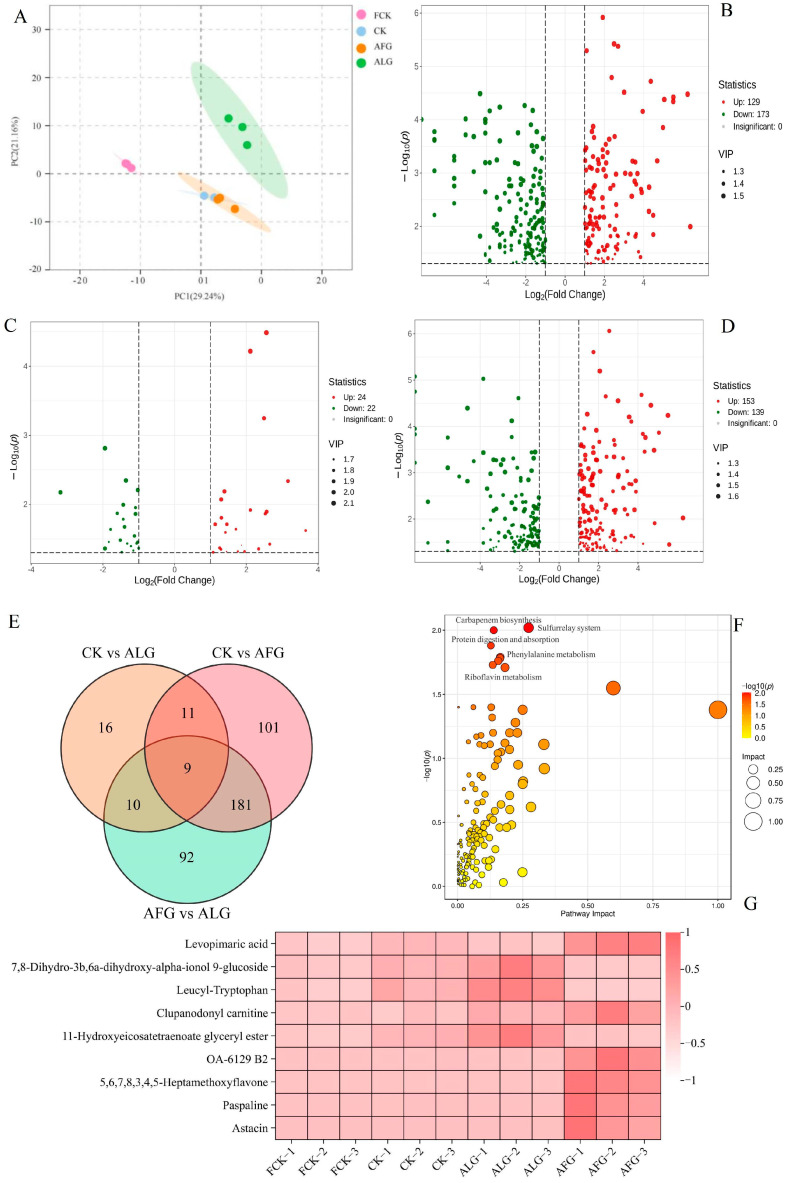
A metabolomic analysis of lamb meatballs. (**A**): PCA plot; (**B**): CK vs. AFG volcano plot; (**C**): CK vs. ALG volcano plot; (**D**): AFG vs. ALG volcano plot; (**E**): Venn diagram of different treatment groups in lamb meatballs; (**F**): Bubble map of KEGG pathways in lamb meatballs; (**G**): Heat map of differential substances in lamb meatballs.

A non-targeted metabolomics analysis of lamb meatballs was conducted on frozen 0 day samples (FCK) and samples from three treatment groups at 30 days, resulting in the identification of 1914 compounds across 15 different classes. Principal Component Analysis (PCA) demonstrated significant differentiation between the samples frozen for 30 days and those at baseline (Figure 4A), with distinct clustering in the 30-day AFG samples compared to CK. Metabolic profiling, guided by the criteria of VIP > 1, *p* ≤ 0.05, and fold change (FC) ≥ 2 or ≤ 0.5, revealed 302 differential metabolites between CK and AFGs; specifically, 129 metabolites were elevated, and 173 were reduced (Figure 4B). Comparatively, 46 differential metabolites were identified between CK and ALG, with 24 increased and 22 decreased (Figure 4C). Between the AFGs and ALG, 292 metabolites differed, comprising 153 that were up-regulated and 139 that were down-regulated (Figure 4D).

Among the three groups, a total of nine shared differential substances were documented (Figure 4E). The variation in the composition of the fresh meat (FCK) and control (CK) was marginal, yet certain metabolites such as 7,8-Dihydro-3b,6a-dih-ydroxy-alpha-ionol 9-glucoside, Leucyl-Tryptophan, and 11-Hydroxyeicosatetraenoate glyceryl ester were markedly declined following AFG treatment and ascended following ALG treatment. Both 7,8-Dihydro-3b,6a-dihydroxy-alpha-ionol 9-glucoside and Leucyl-Tryptophan play roles in lipid and fatty acid metabolism [41], indicating that AFGs mitigate lipid oxidation and postpone the onset of lipid deterioration in lamb meat. Substances such as 7,8-Dihydro-3b,6a-dihydroxy-alpha-ionol 9-glucoside, Leucyl-Tryptophan, OA-6129 B2, 5,6,7,8,3,4,5-Heptamethoxyflavone, Paspaline, and As-tacin were elevated following AFG treatment, with no significant discrepancies observed between the ALG and CK treatments (Figure 4G, Table 1). OA-6129 B2, identified as a carbapenem antibiotic [42], might reflect drug residues inherently present in the mutton.

KEGG pathway analysis revealed enrichment in five pathways: the sulfur relay system, carbapenem biosynthesis, protein digestion and absorption, phenylalanine metabolism, and riboflavin metabolism. Within these, Levopimaric acid, 5,6,7,8,3,4,5-Heptamethoxyflavon, and Paspaline were linked to functions such as apoptosis and signaling in the sulfur relay system pathway (Figure 4F) [43]. Although not directly connected to the sulfur relay system, 11-Hydroxyeicosatetraenoate glyceryl ester, a fatty acid derivative, plays a role in cell signaling and inflammatory responses, processes related to this pathway. The detection of OA-6129 B2 in the samples, which were procured from a local market potentially contaminated with antibiotics during farming, highlights its association with the carbapenem biosynthesis pathway.

Leucyl-Tryptophan and 7,8-Dihydro-3b,6a-dihydroxy-alphaionol 9-glucoside are involved in the pathway of protein digestion and absorption, where proteins are decomposed into peptides and amino acids. While 7,8-Dihydro-3b,6a-dih-ydroxy-alphaionol 9-glucoside is primarily an O-acyl carbohydrate, its involvement may extend to carbohydrate digestion and absorption, influencing energy provision and consequently impacting protein metabolism [44]. Associated with phenylalanine metabolism are Astacin, a metalloprotease potentially engaged in protein degradation, and 7,8-Dihydro-3b,6a-dihydroxy-alphaionol 9-glucoside. The breakdown of proteins releases amino acids, including phenylalanine, thereby indirectly affecting its metabolism [45].

Leucyl-tryptophan pertains to riboflavin metabolism, where tryptophan is crucial, and leucine contributes to protein synthesis and energy metabolism—processes integral to riboflavin’s role as a coenzyme [46]. Similarly, paspaline’s involvement in the riboflavin metabolism pathway may be indirect, possibly through its roles in cell signaling and metabolic regulation [47]. In summary, AFGs effectively decelerate the oxidation and degradation of lamb meat, thereby preserving the flavor and quality of lamb meatballs over an extended period.

## 4. Conclusions

Research demonstrates that antifreeze glycopeptides serve as potent antifreeze agents for lamb meat, potentially enhancing the quality of frozen lamb by suppressing ice crystal formation. This study demonstrated that antifreeze glycopeptides can serve as effective cryoprotectants, mitigating the quality degradation of Xinjiang mutton post-freezing. The application of AFGs effectively mitigated the deterioration of quality in mutton meatballs by postponing increases in TBARS and TVB-N levels, enhancing water retention, averting water migration, and curbing the development of large ice formations during frozen storage. Additionally, AFGs provided protection to the microstructure of muscle tissues and curtailed protein and lipid oxidation. Consequently, employing AFGs as a cryoprotectant can obstruct ice crystal proliferation in mutton meatballs, elevate the quality of mutton, and diminish storage expenses.

Metabolomic analysis revealed that significant changes in the metabolite profiles were caused by AFG treatment. A total of 1914 compounds were identified, with 302 differential metabolites observed between the control (CK) and AFG-treated groups. Key metabolites such as leucyl-tryptophan and 11-Hydroxyeicosatetraenoate glyceryl ester were significantly altered, indicating that AFGs mitigate lipid oxidation and delays the onset of lipid deterioration. These metabolites, which are involved in lipid and fatty acid metabolism, protein digestion, and amino acid metabolism, serve as potential biomarkers for evaluating the cryoprotective effects of AFGs.

In summary, AFGs effectively decelerates the oxidation and degradation of lamb meat, thereby preserving its flavor, texture, and nutritional quality over extended frozen storage. The findings of this study provide a scientific basis for the application of AFGs as a natural and efficient cryoprotectant in the meat industry, offering a promising strategy for enhancing the quality and shelf life of frozen lamb products. Future studies should focus on further validating these metabolites as biomarkers and exploring their correlation with sensory and textural attributes to optimize the use of AFGs in frozen meat preservation.

## Figures and Tables

**Table 1:** List of significantly different substances in lamb meatballs.

No.	Compounds	Formula	Related Category	RT/s	*m/z*	Ionization Mode	CK vs. ALG		CK vs. AFGs		ALG vs. AFGs	
							Trend	*p*	Trend	*p*	Trend	*p*
1	Levopimaric acid	C_20_H_30_O_2_	Others	401	303.2297	pos	up	0.01	up	0.00	up	0.00
2	7,8-Dihydro-3b,6a-dihydroxy-alpha-ionol 9-glucoside	C_19_H_34_O_8_	Lipids and lipid-like molecules	335.7	373.2156	pos	up	0.01	up	0.00	up	0.00
3	Leucyl-Tryptophan	C_17_H_23_N_3_O_3_	Organic acids and derivatives	327.8	356.2785	pos	up	0.01	up	0.00	up	0.00
4	Clupanodonyl carnitine	C_29_H_47_NO_4_	Lipids and lipid-like molecules	362.5	474.352	pos	up	0.00	up	0.03	up	0.01
5	11-Hydroxyeicosatetraenoate glyceryl ester	C_23_H_38_O_5_	Lipids and lipid-like molecules	314	378.3222	pos	up	0.05	up	0.01	up	0.04
6	OA-6129 B2	C_20_H_31_N_3_O_8_S	Others	48.8	472.1824	neg	up	0.01	up	0.00	/	0.00
7	5,6,7,8,3,4,5-Heptamethoxyflavone	C_22_H_24_O_9_	Others	241.2	431.2274	neg	up	0.00	up	0.00	/	0.00
8	Paspaline	C_28_H_39_NO_2_	Others	407.7	466.2907	neg	up	0.02	up	0.00	/	0.00
9	Astacin	C_40_H_48_O_4_	Lipids and lipid-like molecules	391.1	591.3568	neg	up	0.01	up	0.00	/	0.00

## Data Availability

The data presented in this study are available on request from the corresponding author.
